# Prevalence, predictors, and prognostic implications of PR interval prolongation in patients with heart failure

**DOI:** 10.1007/s00392-017-1162-6

**Published:** 2017-09-15

**Authors:** Theodora Nikolaidou, Pierpaolo Pellicori, Jufen Zhang, Syed Kazmi, Kevin M. Goode, John G. Cleland, Andrew L. Clark

**Affiliations:** 1 Department of Academic Cardiology, Castle Hill Hospital, University of Hull, Level 1 Daisy Building, Castle Road, Hull, HU16 5JQ UK; 2 Anglia Ruskin University, Cambridge, UK; 3 National Heart and Lung Institute, Imperial College London and Robertson Centre for Biostatistics and Clinical Trials, University of Glasgow, Glasgow, UK

**Keywords:** First-degree heart block, Heart failure, PR interval

## Abstract

**Aims:**

To determine the prevalence, incidence, predictors, and prognostic implications of PR interval prolongation in patients referred with suspected heart failure.

**Methods and results:**

Consecutive patients referred with suspected heart failure were prospectively enrolled. After excluding patients with implantable cardiac devices and atrial fibrillation, 1420 patients with heart failure and reduced ejection fraction (HeFREF) [age: median 71 (interquartile range IQR 63–78) years; men: 71%; NT-ProBNP: 1319 (583–3378) ng/L], 1094 with heart failure and normal ejection fraction (HeFNEF) [age: 76 (70–82) years; men: 47%; NT-ProBNP: 547 (321–1171) ng/L], and 1150 without heart failure [age: 68 (60–75) years; men: 51%; NT-ProBNP: 86 (46–140) ng/L] were included. The prevalence of first-degree heart block [heart rate corrected PR interval (PRc) > 200 ms] was higher in patients with heart failure (21% HeFREF, 20% HeFNEF, 9% without heart failure). In patients with HeFREF or HeFNEF, longer baseline PRc was associated with greater age, male sex, and longer QRS duration, and, in those with HeFREF, treatment with amiodarone or digoxin. Patients with heart failure in the longest PRc quartile had worse survival compared to shorter PRc quartiles, but PRc was not independently associated with survival in multivariable analysis. For patients without heart failure, shorter baseline PRc was independently associated with worse survival.

**Conclusion:**

PRc prolongation is common in patients with HeFREF or HeFNEF and associated with worse survival, although not an independent predictor of outcome. The results of clinical trials investigating the therapeutic potential of shortening the PR interval by pacing are awaited.

**Electronic supplementary material:**

The online version of this article (doi:10.1007/s00392-017-1162-6) contains supplementary material, which is available to authorized users.

## Introduction

Abnormal myocardial function is often accompanied by disturbances in electrical conduction. Patients with heart failure with reduced ejection fraction (HeFREF) have a substantial prevalence and annual incidence of QRS prolongation [1, 2], both of which are associated with worse outcomes [2, 3]. However, there are few reports on the prevalence, incidence, and prognostic significance of the PR interval in ambulatory patients with chronic heart failure [4–6]. Analyses from studies in the general population have shown that PR interval prolongation is associated with increased risk of atrial fibrillation, pacemaker implantation, and mortality [7–10].

Most available data in heart failure relate to patients undergoing device implantation rather than coming from an unselected population [7]. Kronborg et al. reported that in 659 patients undergoing CRT implantation, 47% had first-degree heart block, and that a long native PR interval was an independent predictor of all-cause and cardiac mortality [11]. In CARE-HF, a longer native PR interval at baseline and longer PR interval at 3 months (paced PR for the CRT group and native PR for the control group) predicted both all-cause mortality and urgent hospitalisation for heart failure even after adjusting for CRT [5]. In patients with advanced heart failure, up to half of arrhythmic deaths may be bradycardic in origin, including high degree atrioventricular block [12–14].

The introduction of cardiac resynchronisation therapy (CRT) has focussed interest on QRS duration and morphology in patients with heart failure. Although CRT was originally conceived by many as a treatment for ventricular dyssynchrony, imaging studies have cast doubt on this hypothesis, leaving considerable doubt about precisely how CRT exerts benefit. Indeed, it may be that the predominant mechanism of benefit of CRT varies from one patient to the next and over time, and might include reduction of atrioventricular delay. Recent reports [6, 15, 16] suggest that PR prolongation, a potential surrogate for greater atrioventricular delay, might also be a target for CRT.

The aim of the present analysis was to describe the prevalence, incidence, predictors, and prognostic implications of a prolonged PR interval in patients with suspected heart failure and, if confirmed, with or without a reduced left ventricular ejection fraction (LVEF).

## Methods

### Ethics approval

The investigation conforms with the principles outlined in the Declaration of Helsinki. It was approved by the Hull and East Yorkshire Research Ethics Committee (Heart Care Study ELSY 2642). All subjects gave written informed consent.

### Participants

Consecutive patients referred with suspected heart failure to a community heart failure clinic between 2001 and 2014 were enrolled. Only patients in sinus rhythm on their baseline electrocardiogram who did not have a ventricular pacing device were included in the analysis. Heart failure with reduced ejection fraction (HeFREF) was defined as the presence of symptoms compatible with the diagnosis of heart failure and impaired left ventricular (LV) systolic function measured as an LVEF < 45% on echocardiography where possible and estimated visually when not. Heart failure with normal ejection fraction (HeFNEF) was defined as the presence of symptoms compatible with the diagnosis of heart failure with LVEF ≥ 45% and, for these patients in sinus rhythm, an amino-terminal pro-B-type natriuretic peptide (NT-ProBNP) ≥ 220 ng/mL. Patients with an LVEF ≥ 45% and an NT-ProBNP < 220 ng/mL were considered not to have heart failure.

Electrocardiographic intervals were obtained from automated analysis of the surface electrocardiogram (ECG). The only exclusion criteria were the inability to provide informed consent, pregnancy, atrial fibrillation, and an implanted cardiac device, even if not pacing at the time of the ECG recording, because the effect that pacing has on native ECG intervals is uncertain. For patients who received a cardiac device during follow-up, survival curves were censored at the time of implant (i.e., the patient was treated as lost to follow-up thereafter). Patients with paroxysmal atrial fibrillation who were in sinus rhythm on the baseline electrocardiogram were included in the analysis.

### Clinical assessment

Baseline characteristics included medical history, therapy, height, weight and blood pressure, blood tests [standard haematology and biochemistry tests, thyroid stimulating hormone (TSH), and NT-ProBNP], and echo- and electrocardiographic data. Echocardiographic information included LVEF and the presence and severity of mitral regurgitation. Patients with diabetes included those managed by diet-alone or with insulin or other hypoglycaemic agents. Patients with the previous myocardial infarction, coronary artery bypass surgery, or positive tests for ischaemia were considered to have ischaemic heart disease (IHD). Cerebrovascular disease was defined as patients with a history of ischaemic stroke or transient ischaemic attack. The estimated glomerular filtraion rate (eGFR) was calculated using the abbreviated modification of the diet in renal disease (MDRD) equation [17].

### Definition of PRc

Automated 12-lead ECG interval measurements were used to measure heart rate, PR, QRS, and QT intervals. PR interval reported was the longest measured PR interval in any lead. The PR interval duration changes with heart rate (longer PR interval at faster heart rates). Heart rate changes continually, therefore, to compare PR intervals taken at different heart rates, an adjustment is made (PRc) using a previously published formula [18]. We used PRc throughout our analysis, except in the hazard ratio analysis, where baseline heart rate is independently associated with worse survival in some groups, and for this reason, we present heart rate and unadjusted PR as separate variables. QT interval was also corrected for heart rate using Bazett’s formula [19]. First-degree heart block was defined as PRc interval > 200 ms.

### Follow-up

Follow-up for all patients was censored at the last point of contact in primary or secondary care to avoid unreported deaths due, for instance, to emigration out of the region. Patients diagnosed with HeFREF were routinely given follow-up appointments at 4 and 12 months with other visits as clinically indicated. Patients in whom the diagnosis of heart failure was excluded, as well as many patients with HeFNEF, were discharged and followed up only through electronic primary and secondary care health records which usually did not include electrocardiographic data. Accordingly, electrocardiographic data during follow-up were only available for the patients with HeFREF.

### Statistical analysis

Data are presented as median and interquartile range (IQR). Categorical data are presented as numbers and percentages. Differences between two independent groups of continuous data were tested using the independent *t* test. The Chi-square test was used for comparisons between independent groups of categorical data and a one-way ANOVA was used to compare more than two independent groups of continuous data. For paired samples, the paired *t* test was used for continuous data. For categorical data with two or more categories, the McNemar’s test and the marginal homogeneity non-parametric test were used, respectively. For comparisons across PRc quartiles, trend statistical tests were used. SPSS (version 22) statistical computer package was used for data analysis.

The distribution of NT-ProBNP and TSH was skewed; we, therefore, used non-parametric tests to compare groups of continuous data and log-transformed NT-ProBNP and TSH data to satisfy the assumptions of the models.

Pearson correlation coefficients were used to observe the correlation between baseline variables in patients with HeFREF. Multiple linear regressions were used to identify variables associated with PRc interval and the Cox regression model was used to identify the variables associated with all-cause mortality. Only the significant variables identified using univariable analysis were used in the multivariable Cox regression models. We used two-tailed tests at a significance level of 0.05.

## Results

**Fig. 1:**
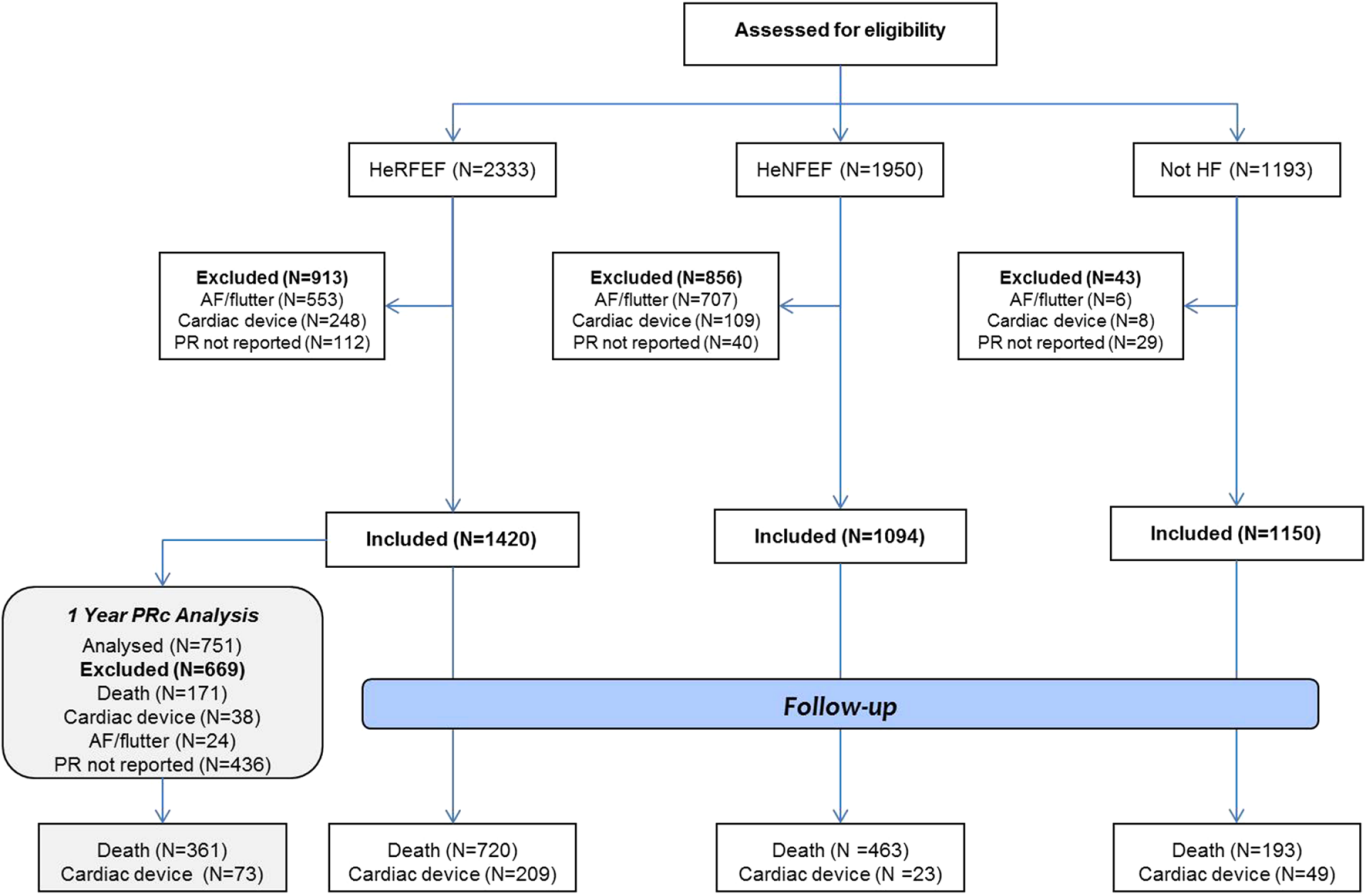
Patient eligibility flow diagram

Of 2333 patients with HeFREF, 1950 with HeFNEF, and 1193 without heart failure, respectively, 913 (39%), 856 (44%), and 43 (4%) were excluded, mainly because of atrial fibrillation or an implanted device (Fig. 1). This left 1,420 patients with HeFREF, 1094 with HeFNEF, and 1150 without heart failure for the analysis (Table 1). Patients with HeFREF were younger, more likely to be men and had lower body surface area compared to those with HeFNEF. Patients without heart failure were slightly younger than in the other two groups. On average, compared to those with HeFNEF, patients with HeFREF had a faster heart rate, longer PRc, QRS, and QTc intervals and lower systolic and diastolic blood pressures, but eGFR and plasma TSH and mortality at 12 months were similar.

**Table 1:** Baseline demographic and clinical characteristics

	Missing *N*	HeFREF (*N* = 1420)	HeFNEF (*N* = 1094)	Not HF (*N* = 1150)	*P* value HeFREF vs. Not HF	*P* value HeFREF vs. HeFNEF	*P* value HeFNEF vs. Not HF	*P* value All groups
Age (years)	–	71 (63–78)	76 (70–82)	68 (60–75)	**< 0.001**	**< 0.001**	**< 0.001**	**< 0.001**
Men (%)	–	1005 (71)	516 (47)	584 (51)	**< 0.001**	**< 0.001**	0.156	**< 0.001**
NYHA class (%)	2/17/161							
I		212 (15)	325 (30)	488 (49)	**< 0.001**	**< 0.001**	**< 0.001**	**< 0.001**
II		708 (50)	493 (46)	369 (37)				
III		457 (32)	244 (23)	124 (13)				
IV		41 (3)	15 (1)	8 (1)				
Diabetes (%)	2/3/4	356 (25)	287 (26)	259 (23)	0.12	0.36	0.06	0.13
Ischaemic heart disease (%)	2/7/15	932 (66)	476 (44)	244 (22)	**< 0.001**	**< 0.001**	**< 0.001**	**< 0.001**
Cerebrovascular disease (%)	2/7/15	100 (7)	68 (6)	40 (4)	**< 0.001**	**< 0.001**	**0.01**	**0.001**
Body surface area (m^2^)	6/7/4	1.91 (1.73–2.08)	1.85 (1.71–2.04)	1.95 (1.78–2.11)	**< 0.001**	**0.01**	**< 0.001**	**< 0.001**
Systolic BP (mmHg)	13/3/21	129 (114–147)	149 (131–167)	147 (132–162)	**< 0.001**	**< 0.001**	0.06	**< 0.001**
Diastolic BP (mmHg)	13/3/21	76 (66–84)	78 (70–88)	83 (75–91)	**< 0.001**	**< 0.001**	**< 0.001**	**< 0.001**
Heart rate (bpm)	–	73 (63–86)	68 (59–78)	69 (60–79)	**< 0.001**	**< 0.001**	0.18	**< 0.001**
QRS (ms)	6//1/4	112 (96–140)	92 (84–106)	90 (82–98)	**< 0.001**	**< 0.001**	**< 0.001**	**< 0.001**
QRS ≥ 150 ms (%)	6//1/4	249 (18)	51 (5)	18 (2)	**< 0.001**	**< 0.001**	**< 0.001**	**< 0.001**
PR (ms)	–	172 (154–194)	168 (152–192)	162 (148–180)	**< 0.001**	0.53	**< 0.001**	**< 0.001**
PRc (ms)	–	174 (157–196)	168 (151–192)	163 (147–179)	**< 0.001**	0.03	**< 0.001**	**< 0.001**
QT (ms)	19/19/6	413 (380–446)	406 (384–431)	396 (372–416)	**< 0.001**	**< 0.001**	**< 0.001**	**< 0.001**
QTc (ms)	19/19/6	453 (422–483)	429 (410–452)	418 (401–441)	**< 0.001**	**< 0.001**	**< 0.001**	**< 0.001**
TSH (mIU/L)	117/91/196	1.6 (1.0–2.5)	1.7 (1.1–2.5)	1.6 (1.0–2.3)	**0.04**	0.37	**0.01**	**0.02**
eGFR (1.73 mL/min/m^2^)	3/2/12	62 (47–77)	61 (47–75)	75 (63–88)	**< 0.001**	0.15	**< 0.001**	**< 0.001**
NT-ProBNP (ng/L)	436/8/139	1319 (583–3378)	547 (321–1171)	86 (46–140)	**< 0.001**	**< 0.001**	**< 0.001**	**< 0.001**
Ejection fraction by Simpson’s	564/523/491	33 (27–37)	54 (48–61)	59 (54–64)	**< 0.001**	**< 0.001**	**< 0.001**	**< 0.001**
LV systolic dysfunction (%)	–							
Normal–trivial		0	820 (75)	1151 (100)	**< 0.001**	**< 0.001**	**< 0.001**	**< 0.001**
Mild		0	274 (25)	0				
Mild–moderate		849 (60)	0	0				
>Moderate		571 (40)	0	0				
Mitral regurgitation > mild (%)	27/17/161	447 (32)	124 (13)	27 (2)	**< 0.001**	**< 0.001**	**< 0.001**	**< 0.001**
Medication at initial visit								
β-blocker (%)	14/18/55	862 (61)	550 (51)	285 (26)	**< 0.001**	**< 0.001**	**< 0.001**	**< 0.001**
ACE-I (%)	14/18/55	1019 (72)	532 (49)	325 (30)	**< 0.001**	**< 0.001**	**< 0.001**	**< 0.001**
ARB (%)	14/18/55	133 (9)	161 (15)	128 (12)	0.07	**< 0.001**	**0.02**	**< 0.001**
MRA (%)	14/18/55	422 (30)	95 (9)	22 (2)	**< 0.001**	**< 0.001**	**< 0.001**	**< 0.001**
Amiodarone (%)	6/18/36	66 (5)	19 (2)	6 (0.5)	**< 0.001**	**0.001**	**< 0.001**	**< 0.001**
Digoxin no. (%)	6/18/36	118 (8)	40 (4)	9 (0.8)	**< 0.001**	**< 0.001**	**< 0.001**	**< 0.001**
Loop Diuretic (%)	8/18/3	993 (70)	557 (52)	249 (22)	**< 0.001**	**< 0.001**	**< 0.001**	**< 0.001**
Ivabradine (%)	14/18/55	5 (0.4)	2 (0.2)	0	0.07	0.35	0.5	0.14
1 year mortality (%)	–	171 (12)	76 (10)	8 (1)	**< 0.001**	0.13	**< 0.001**	**< 0.001**

Bold indicates significance at the 0.05 level

Continuous variables are presented as median (interquartile range), whereas categorical variables are expressed as percentage. *P* values are for differences between patients with HeFREF, HeFNEF, and those without heart failure. Pair-wise comparisons were performed using the independent *t* test for continuous data (except for TSH and NT-ProBNP where the Mann–Whitney test was used) and the Chi-square test for categorical data. The one-way ANOVA test was used for comparisons of continuous data across all groups (except for TSH and NT-ProBNP where the Kruskal–Wallis test was used) and the Chi-square test for categorical data

*ACE-I* angiotensin converting enzyme inhibitor, *ARB* angiotensin receptor blocker, *BP* blood pressure, *eGFR* estimated glomerular filtration rate, *MRA* mineralocorticoid receptor antagonist, *NYHA* New York Heart Association, *TSH* thyroid stimulating hormone

Amongst patients with HeFREF, 21% had a PRc > 200 ms compared to 20% of patients with HeFNEF (*P* = 0.33) and 9% of those without heart failure (*P* < 0.001) (Supporting Fig. 1A), but the proportions with a PRc > 230 ms were substantially lower, 5, 7, and 2%, respectively. With increasing quartile of PRc duration, in each diagnostic group, a higher proportion of patients were aged > 65 years and men and median QRS duration increased (Table 2 and Supporting Tables 1, 2). Patients with HeFREF and longer PRc were also in a worse New York Heart Association class, which had worse left ventricular systolic function and greater use of loop diuretics (Table 2). Patients with HeFNEF and longer PRc had greater use of loop diuretics (Supporting Table 1).

**Table 2:** Baseline demographic and clinical characteristics of patients with HeFREF, classified by PRc quartiles

	PRc Q1 *N* = 355	PRc Q2 *N* = 355	PRc Q3 *N* = 355	PRc Q4 *N* = 355	*P* value
Age (years)	70 (61–77)	70 (61–77)	71 (63–78)	73 (65–79)	**< 0.001**
Men, no. (%)	225 (63)	236 (67)	259 (72)	285 (81)	**< 0.001**
NYHA class, no. (%)					
I	59 (17)	51 (14)	54 (15)	47 (13)	**0.002**
II	193 (54)	180 (51)	171 (48)	164 (46)	
III	96 (27)	114 (32)	122 (35)	125 (36)	
IV	7 (2)	9 (3)	6 (2)	19 (5)	
Diabetes, no. (%)	64 (18)	83 (23)	96 (27)	113 (32)	**0.001**
Ischaemic heart disease, no. (%)	232 (65)	232 (65)	228 (64)	240 (68)	0.64
Cerebrovascular disease, no. (%)	17 (5)	30 (8)	28 (8)	28 (8)	0.19
Body surface area (m^2^)	1.86 (1.67–2.05)	1.90 (1.71–2.08)	1.91 (1.75–2.07)	1.95 (1.78–2.11)	**< 0.001**
Systolic BP (mmHg)	130 (115–147)	129 (115–147)	130 (115–147)	128 (112–147)	0.69
Diastolic BP (mmHg)	75 (67–83)	75 (66–84)	78 (69–87)	75 (65–83)	0.89
Heart rate (bpm)	70 (60–84)	74 (64–87)	72 (62–86)	73 (62–86)	0.34
QRS (ms)	104 (92–134)	106 (96–136)	114 (98–142)	120 (104–148)	**< 0.001**
QRS ≥ 150 ms (%)	44 (12)	51 (14)	69 (19)	88 (25)	**< 0.001**
PR (ms)	144 (134–151)	164 (160–168)	182 (176–188)	210 (200–222)	–
PRc (ms)	146 (136–152)	166 (162–170)	184 (178–189)	212 (203–224)	–
QT (ms)	417 (382–446)	406 (374–440)	412 (385–449)	418 (384–452)	0.15
QTc (ms)	447 (419–478)	453 (421–482)	454 (422–486)	456 (424–484)	**0.01**
Thyroid stimulating hormone (mIU/L)	1.5 (1.0–2.4)	1.5 (1.0–2.3)	1.7 (1.0–2.6)	1.7 (1.1–2.7)	**0.01**
eGFR (1.73 mL/min/m^2^)	66 (50–81)	63 (48–79)	61 (46–77)	58 (44–71)	**< 0.001**
NT-ProBNP (ng/L)	1311 (582–3045)	1048 (446–3331)	1400 (548–3350)	1751 (811–3915)	**0.50**
Ejection fraction by Simpson’s	32 (27–38)	33 (27–38)	33 (27–37)	32 (25–37)	0.19
Left ventricular dysfunction, no. (%)					**0.02**
Normal–trivial	–	–	–	–	
Mild	0	0	0	0	
Mild–moderate	227 (64)	213 (60)	213 (60)	196 (55)	
>Moderate	128 (36)	142 (40)	142 (40)	159 (45)	
Mitral regurgitation > mild	108 (31)	100 (29)	124 (36)	115 (33)	0.23
β-blocker, no. (%)	208 (60)	222 (63)	214 (61)	218 (61)	0.57
ACE-I, no. (%)	259 (74)	254 (72)	251 (71)	255 (73)	0.72
ARB, no. (%)	31 (9)	37 (10)	26 (7)	39 (11)	0.59
MRA, no. (%)	87 (25)	109 (31)	115 (32)	111 (32)	**0.05**
Amiodarone, no. (%)	14 (4)	9 (3)	14 (4)	29 (8)	**0.005**
Digoxin, no. (%)	20 (6)	28 (8)	32 (9)	38 (11)	**0.01**
Loop diuretic, no. (%)	234 (67)	237 (67)	246 (70)	276 (78)	0.001
Ivabradine, no. (%)	3 (0.8)	1 (0.3)	0	1 (0.3)	0.16
1 year mortality, no. (%)	36 (10)	49 (14)	36 (11)	49 (13)	0.34

Bold indicates significance at the 0.05 level

Continuous variables are presented as median (interquartile range), whereas categorical variables are expressed as numbers (percentage). *P* values are for differences between PRc quartiles (columns 2, 3, 4, and 5). The one-way ANOVA linear trend test was used for comparisons of continuous data across groups and the Cochran’s Chi-square trend test for categorical data

*ACE-I* angiotensin converting enzyme inhibitor, *ARB* angiotensin receptor blocker, *BP* blood pressure, *eGFR* estimated glomerular filtration rate, *MRA* mineralocorticoid receptor antagonist, *NYHA* New York Heart Association. See also Supporting Tables 1 and 2

### Variables associated with a long PR interval

In all diagnostic groups, heart rate was inversely related to QT, PR, and QRS intervals (Supporting Table 3). PRc correlated weakly with QRS (*R* ^2^ = 0.04; *P* < 0.001) and to a lesser extent with QTc duration (*R* ^2^ = 0.005; *P* = 0.01) but not heart rate (Supporting Table 3).

**Table 3:** Univariable and multivariable analyses of variables associated with baseline PRc in patients with HeFREF, HeFNEF, and those without heart failure

		Univariable model			Multivariable model								
		HeFREF *N* = 1420	HeFNEF *N* = 1094	No HF *N* = 1150	HeFREF (*R* ^2^ = 0.12) *N* = 976			HeFNEF (*R* ^2^ = 0.15) *N* = 1046			Not HF (*R* ^2^ = 0.16) *N* = 884		
		*P*		*P*	Unstandardized coefficient *B* (95% CI)	*P*	Standardized coefficient	Unstandardized coefficient *B* (95% CI)	*P*	Standardized coefficient	Unstandardized coefficient *B* (95% CI)	*P*	Standardized coefficient
Age	Per decade increase	**< 0.001**	**< 0.001**	**< 0.001**	4.94 (2.87, 7.01)	**< 0.001**	0.18	8.83 (6.30, 11.36)	**< 0.001**	0.23	4.79 (2.88, 6.71)	**< 0.001**	0.19
Sex	Men vs. women	**< 0.001**	**< 0.001**	**< 0.001**	10.41 (5.64, 15.17)	**< 0.001**	0.15	8.83 (4.16, 14.01)	**< 0.001**	0.12	2.57 (−1.42, 6.56)	0.20	0.05
QRS	Per 10 ms increase	**< 0.001**	**< 0.001**	**< 0.001**	1.53 (0.86, 2.21)	**< 0.001**	0.14	2.64 (1.78, 3.510)	**< 0.001**	0.18	2.75 (1.67, 3.83)	**< 0.001**	0.17
BSA	Per m^2^ increase	**< 0.001**	**< 0.001**	**< 0.001**	16.86 (7.14, 26.57)	**0.001**	0.14	23.68 (13.33, 34.03)	**< 0.001**	0.17	19.27 (11.27, 27.27)	**< 0.001**	0.17
Diabetes	Yes vs. no	**< 0.001**	0.20	**< 0.001**	3.73 (−0.88, 8.35)	0.13	0.05	1.14 (−3.73, 6.02)	0.65	0.01	6.52 (2.43, 10.61)	**0.002**	0.10
NYHA	III/IV vs. I/II	**0.004**	0.08	0.34	2.86 (−1.31, 7.04)	0.17	0.04	1.84 (−3.25, 6.94)	0.48	0.02	1.33 (−3.65, 6.31)	0.54	0.02
Log[NT-ProBNP]	Per Log[NT-ProBNP] increase	**0.04**	**0.01**	0.56	2.10 (−1.79, 6.00)	0.27	0.04	4.06 (−1.65, 9.77)	0.16	0.05	−4.42 (−10.27, 1.43)	0.14	−0.05
Systolic BP	Per 5 mmHg increase	0.89	0.94	0.01	−0.20 (−0.63, 0.23)	0.38	−0.03	0.04 (−0.38, 0.45)	0.86	0.01	0.10 (−0.33, 0.52)	0.64	0.02
eGFR	Per 5 mL/min/1.73 m^2^	**< 0.001**	**0.01**	**< 0.001**	0.12 (−0.46, 0.69)	0.70	0.01	0.13 (−0.46, 0.72)	0.67	0.01	−0.79 (−1.41, −0.17)	**0.01**	−0.09
Digoxin	Yes vs. no	**0.01**	0.08	0.32	10.36 (3.12, 17.59)	0.01	0.09	6.69 (−4.63, 18.01)	0.25	0.03	9.72 (−8.96, 28.40)	0.31	0.04
Amiodarone	Yes vs. no	**< 0.001**	0.07	0.94	10.94 (1.93, 19.95)	**0.01**	0.08	−2.29 (−17.84, 13.26)	0.77	−0.01	−11.24 (−34.22, 11.73)	0.37	−0.03
Loop diuretic	Yes vs. no	**0.03**	**0.049**	0.09	2.85 (−1.53, 7.23)	0.18	0.04	−2.60 (−7.19, 2.00)	0.27	−0.04	−1.35 (−5.48, 2.78)	0.52	−0.02
ARB	Yes vs. no	**0.04**	0.41	0.08	2.42 (−5.03, 9.87)	0.51	0.02	2.87 (−3.32, 9.05)	0.36	0.03	−2.05 (−7.41, 3.31)	0.45	−0.03
MRA	Yes vs. no	**0.02**	0.23	**0.03**	0.60 (−3.93, 5.14)	0.79	0.01	2.31 (−5.41, 10.04)	0.56	0.02	5.69 (−5.22, 17.12)	0.29	0.03
ACE-I	Yes vs. no	0.27	0.23	**0.003**	−0.54 (−5.62, 4.55)	0.85	−0.01	1.14 (−3.41, 5.69)	0.62	0.02	2.02 (−1.39, 6.53)	0.20	0.04
β-blocker	Yes vs. no	0.70	**0.003**	**0.003**	−0.47 (−0.4.56, 3.63)	0.82	−0.01	4.68 (0.41, 8.95)	0.03	0.06	4.78 (0.80, 8.77)	**0.02**	0.08

Bold indicates significance at the 0.05 level

Linear regression of the probability of a longer PRc interval is based on clinical characteristics and drug therapy. Variables are ranked by standardized coefficient of the multivariable analysis for patients with HeFREF. A negative standardized coefficient reflects a negative association

*ACE-I* angiotensin converting enzyme inhibitor, *ARB* angiotensin receptor blocker, *BP* blood pressure, *eGFR* estimated glomerular filtration rate, *LV* left ventricular, *MRA* mineralocorticoid receptor antagonist, *NYHA* New York Heart Association

In patients without heart failure, longer PRc was positively correlated with a history of paroxysmal atrial fibrillation (1% had paroxysmal atrial fibrillation in the lowest PRc quartile compared to 5% in the highest PRc quartile; *P* = 0.03; data not shown). This association was not present in patients with heart failure.

In a multivariable linear regression model, in patients with HeFREF, longer PRc interval was associated with greater age and male sex, longer QRS duration, and prescription of digoxin or amiodarone (Table 3). In patients with HeFNEF, longer PRc interval was associated with greater age and male sex, longer QRS duration, and prescription of β-blockers (Table 3). In patients without heart failure, longer PRc interval was associated with greater age and diabetes, longer QRS duration, lower eGFR, and prescription of β-blockers.

### One-year follow-up in patients with HeFREF

Paired assessments at baseline and 1 year were available for 751 patients with HeFREF (Supporting Table 4); 171 had died, 24 developed atrial fibrillation, 38 had an implanted device, and 436 did not have a repeat electrocardiogram. Twenty-seven patients who developed AF at 1 year had an average baseline PRc of 179 ms, while the 38 patients requiring implantation of any cardiac device had an average PRc of 184 ms.

**Table 4:** Hazard ratios for mortality from baseline characteristics in patients with HeFREF, HeFNEF, and those without heart failure

Cox regression		HeFREF *N* = 975			HeFNEF *N* = 1040			Not HF *N* = 873		
Variable	Hazard ratio representation	Hazard ratio (95% CI)	*P* value	Wald	Hazard ratio (95% CI)	*P* value	Wald	Hazard ratio (95% CI)	*P* value	Wald
Log[NT-ProBNP]	Per Log[NT-ProBNP] increase	2.23 (1.78, 2.77)	**< 0.001**	50.27	2.12 (1.63, 2.75)	**< 0.001**	31.39	1.91 (0.98, 3.73)	**0.06**	3.56
Age	Per decade increase	1.44 (1.26, 1.65)	**< 0.001**	28.01	1.69 (1.48, 1.95)	**< 0.001**	55.10	1.64 (1.33, 2.02)	**< 0.001**	21.58
Diastolic BP	Per 5 mmHg increase	0.91 (0.87, 0.95)	**< 0.001**	20.53	0.99 (0.95, 1.03)	0.58	0.31	0.94 (0.87, 1.00)	0.06	3.53
NYHA	III/IV vs. I/II	1.39 (1.12, 1.72)	**0.003**	8.95	1.45 (1.15, 1.83)	**0.001**	10.12	1.80 (1.19, 2.73)	**0.006**	7.68
Digoxin	Yes vs. no	1.45 (1.02, 2.07)	**0.03**	4.33	1.78 (1.17, 2.71)	**0.01**	7.32	4.05 (1.67, 9.80)	**0.002**	9.60
eGFR	Per 5 mL/min/1.73 m^2^ increase	0.97 (0.94, 1.00)	**0.03**	4.67	0.99 (0.96, 1.02)	**0.43**	0.62	0.99 (0.93, 1.05)	0.66	0.19
β-blocker	Yes vs. no	0.85 (0.68, 1.07)	0.16	1.97	0.82 (0.65, 1.03)	0.09	2.87	0.61 (0.37, 1.00)	0.05	3.84
Loop diuretic	Yes vs. no	1.21 (0.95, 1.55)	0.13	2.30	1.69 (1.35, 2.11)	**< 0.001**	21.52	1.59 (1.12, 2.27)	**0.01**	6.48
Ischaemic heart disease	Yes vs. no	1.17 (0.93, 1.46)	0.17	1.85	0.90 (0.73, 1.12)	0.34	0.90	1.17 (0.79, 1.72)	0.44	0.60
Body surface area	Per m^2^ increase	0.62 (0.35, 1.10)	0.10	2.69	0.52 (0.31, 0.87)	**0.01**	6.08	0.75 (0.33, 1.67)	0.47	0.51
Sex	Men vs. women	1.16 (0.90, 1.50)	0.26	1.26	1.61 (1.27, 2.03)	**< 0.001**	15.70	2.17 (1.48, 3.21)	**< 0.001**	15.59
Diabetes	Yes vs. no	1.16 (0.90, 1.50)	0.25	1.32	1.38 (1.08, 1.76)	**0.01**	6.83	1.22 (0.77, 1.92)	0.38	0.76
PR	Per 10 ms increase	1.01 (0.98, 1.05)	0.40	0.70	0.99 (0.96–1.03)	0.69	0.16	0.92 (0.85, 0.98)	**0.02**	5.69
Baseline heart rate	Per 10 beats per minute increase	1.03 (0.96, 1.10)	0.48	0.51	1.17 (1.08, 1.26)	**< 0.001**	15.12	1.17 (1.03, 1.33)	**0.02**	5.42
Amiodarone	Yes vs. no	1.13 (0.71, 1.79)	0.61	0.26	1.55 (0.84, 2.86)	0.16	1.94	1.99 (0.60, 6.70)	0.26	1.26
QRS	Per 10 ms increase	0.99 (0.96, 1.03)	0.77	0.09	1.01 (0.96, 1.05)	0.76	0.09	0.96 (0.86, 1.08)	0.47	0.50
CVA/TIA	Yes vs. no	1.02 (0.71, 1.46)	0.92	0.01	1.10 (0.77, 1.58)	0.60	0.28	1.55 (0.79, 3.03)	0.20	1.63

Bold indicates significance at the 0.05 level

Variables ranked by Wald score. Left ventricular dysfunction removed from model due to high correlation with NT-ProBNP

*BP* blood pressure, *CVA/TIA* cerebrovascular accident or transient ischaemic attack, *DBP* diastolic blood pressure; *eGFR* estimated glomerular filtration rate

After 1 year, more patients were on heart failure medication compared to baseline, and LVEF had risen, New York Heart Association class and LV function had improved, NT-ProBNP had declined, and systolic and diastolic blood pressures had fallen. There was a decline in eGFR. Median PRc interval increased slightly further over 1 year [173 ms (156–193) to 176 ms (158–197); *P* < 0.001; Supporting Table 4]. The prevalence of first-degree heart block was similar at baseline (17%) and 1 year (20%; *P* = 0.33); 39 patients with first-degree heart block at baseline had normal values by 1 year, while 52 patients developed new first-degree heart block. PRc duration increased by ≥ 5% in 262 (35%) patients with HeFREF, while it decreased by ≥ 5% in 161 (21%) (Supporting Fig. 1B-C). Change by more than one quartile was rare (Supporting Fig. 1C). Only patients with a documented ECG at 1 year were included in this analysis. This may have resulted in selection bias.

### Survival in patients with HeFREF

**Fig. 2:**
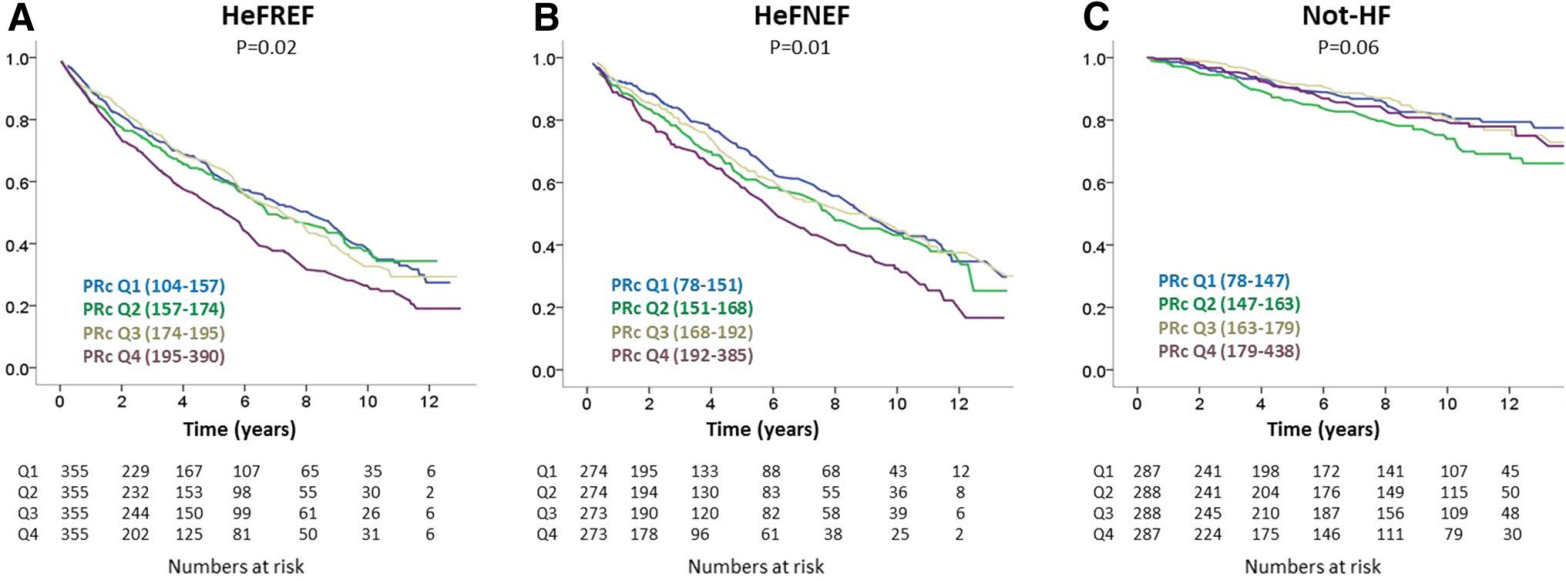
Survival in patients with HeFREF (**a**), HeFNEF (**b**), and without heart failure (**c**) according to baseline PRc quartile (PRc ranges shown in brackets are in milliseconds)

During a median follow-up of 3.9 years (IQR: 1.6–7.1), with follow-up censored at the time of death, 627 (44%; ~11% per annum) patients died (Fig. 2a). Patients in the highest baseline PRc quartile had a worse survival compared to all other quartiles. In a Cox regression model, baseline variables independently associated with worse survival were: increasing log[NT-ProBNP], age, and NYHA class; and decreasing diastolic blood pressure and eGFR and the use of digoxin but neither PR nor PRc (Table 4).

Survival was worse in those with first-degree heart block in the second and subsequent years of follow-up especially if PRc increased between baseline and the first year (Supporting Fig. 2). β-blockers were started in 209 patients between baseline and 1 year. Of these, 46 had first-degree heart block at baseline and a further 24 (12%) developed it by 1 year. There was no interaction between commencing β-blockers, new first-degree heart block at 1 year and survival.

### Survival in patients with HeFNEF

During a median follow-up of 3.7 years (IQR: 1.7–7.4), with follow-up censored at the time of death, 444 (41%; ~11% per annum) patients died. Patients in the highest baseline PRc quartile had a worse survival compared to all other quartiles (Fig. 2b). In a Cox regression model, baseline variables independently associated with worse survival in HeFNEF were: increasing log[NT-ProBNP], male sex, higher New York Heart Association class, and age. Neither PR nor PRc was independent predictors of outcome. The presence of diabetes, a lower body surface area, and the use of a loop diuretic or digoxin were also associated with worse survival (Table 4). A slower baseline heart rate was associated with better survival.

### Survival in patients without heart failure

During a median follow-up of 7.9 years (IQR 3.2–11.2), only 190 (17%; 2% per annum) patients died. Follow-up was much longer than in the heart failure groups because of low mortality leading to low rates of censorship due to death. On an unadjusted analysis of quartiles, shorter PRc and a faster heart rate were independently associated with worse survival (*P* = 0.02 each). Increasing log[NT-ProBNP], male sex and increasing age were associated with worse survival in patients without heart failure as was worse functional class (132 patients in NYHA III/IV) and the use of loop diuretics (249 patients). Of patients taking loop diuretics (249 patients), 73 died (29%; 4% per annum). Of patients with an NT-ProBNP between 125 and 220 ng/L (310 patients), 80 died (26%; 3% per annum). Many patients had chronic lung disease accounting for symptoms.

### Device implantation

During follow-up, 144 patents with HeFREF were implanted with CRT with or without a defibrillator. Twenty-nine patients were implanted with defibrillator alone, while 36 patients had a pacemaker. Thirty-six patients with HeFNEF required a pacemaker during follow-up as did 20 patients without heart failure. Increasing PR and QRS interval duration as well as advancing age were associated with a greater risk of pacemaker implantation (Supporting Table 5).

## Discussion

This study is the first large epidemiological study to report the prevalence and predictors of a long PR interval in patients with heart failure. We found that modest increases in PR duration are common in heart failure with or without a reduced LVEF.

The prevalence of first-degree heart block in patients with HeFREF was 21%. In the subgroup of patients with moderate–severe LV dysfunction and QRS ≥ 130 ms (*N* = 227), the prevalence on first-degree heart block was 29%. This is consistent with the previous studies in patients with heart failure, LVEF < 35%, and left bundle branch block undergoing CRT, where the prevalence of first-degree heart block was reported to be between 26–52% [5, 6, 20]. Amongst patients with HeFNEF and QRS ≥ 130 ms (*N* = 115), the prevalence of first-degree heart block was even higher (40%).

A long PR interval in heart failure is likely to reflect widespread electrophysiological abnormalities, including atrial enlargement and myocardial fibrosis, atrioventricular nodal conduction delay and/or bundle branch/Purkinje fibre conduction delay, altered autonomic tone, and the effects of pharmacological interventions [21]. Studies of animal models of heart failure have shown anatomical and ion channel changes in the atria and atrioventricular node associated with delayed atrioventricular conduction [22].

PRc was not an independent predictor of outcome in patients with heart failure. This adds to existing evidence on first-degree heart block in other populations. Data from the Finish Social Insurance Institution’s Coronary Heart Disease (CHD) Study showed no increased risk of mortality, hospitalisation, or incidence of atrial fibrillation, heart failure, or stroke with first-degree heart block in the general population [23]. In contrast, the Framingham study reported an increased risk of atrial fibrillation, pacemaker implantation, and death with PR interval prolongation at 20 years’ follow-up [8]. In patients with stable coronary artery disease and normal ejection fraction, first-degree heart block was associated with increased mortality and hospitalisation for heart failure in one study [24].

Patients with heart failure and first-degree heart block undergoing CRT have worse prognosis compared to those with a normal PR interval [25, 26]. However, patients with first-degree heart block may derive greater benefit from CRT compared to pharmacological therapy [15]. In the COMPANION trial, a PR interval > 200 ms was associated with a 41% increased risk of the composite outcome of all-cause mortality or heart failure hospitalisation in those assigned to pharmacological therapy but was not higher amongst patients assigned to CRT [6]. A post hoc analysis from MADIT-CRT suggested that patients who did not have LBBB but did have a long PR interval benefited from CRT, whereas those with a normal PR interval did not [15]. The HOPE-HF trial is currently testing whether CRT with atrioventricular optimisation and His bundle pacing is beneficial in patients with heart failure and long PR interval without left bundle branch block [27].

In patients without heart failure, a short rather than a long PRc independently predicted increased mortality. This is in keeping with the previous findings in healthy population studies and in patients with coronary artery disease, suggesting that a short PR interval may not be a benign finding [28, 29]. It is unknown whether a short PR interval is a marker of underlying cardiac pathology (such as the presence of a concealed accessory pathway) or impaired autonomic/electrotonic regulation in the atrioventricular node.

Prolongation of QRS is common in patients with heart failure and is associated with worse outcomes [2, 3]. We found that in patients with heart failure (with or without reduced ejection fraction), neither QRS nor PRc interval duration was independently associated with all-cause mortality in models that included powerful prognostic markers such as age and NT-ProBNP. In the Korean heart failure registry (*N* = 1986), 16% of patients presenting with acute heart failure had first-degree atrioventricular block. The combination of first-degree atrioventricular block with a QRS duration ≥ 120 ms was associated with an increased risk of adverse outcomes, including all-cause mortality [4]. In our study, patients with PRc ≥ 200 ms and QRS ≥ 130 ms had worse survival compared to other groups (Supporting Fig. 3), but combined PRc/QRS duration was not an independent predictor of mortality. Our study shows that similar to data from the general population [8], a long PR interval is associated with higher risk of simple pacemaker implantation in patients with heart failure who do not meet criteria for CRT or defibrillator.

β-blockade, amiodarone, and digoxin all prolong atrioventricular conduction. Patients on amiodarone are likely to have experienced prior atrial or ventricular arrhythmias, and may be more likely to have severe underlying heart disease and conduction abnormalities. However, β-blockade was not associated with prolongation of the PRc interval in patients with HeFREF; the atrioventricular node may be insensitive to β-blockade-induced conduction delay in heart failure, possibly due to down-regulation of adrenergic receptors or a reduction in parasympathetic tone.

A long PR interval is associated with an increased risk of subsequently developing atrial fibrillation in the general population [30]. Our data support this finding in patients without heart failure, in whom longer PRc was positively correlated with a history of paroxysmal atrial fibrillation (1% in the first quartile of PRc had paroxysmal atrial fibrillation compared to 5% in the fourth quartile; *P* = 0.03; data not shown).

### Study limitations

We used a single electrocardiogram to measure PR interval. The PR interval varies during the day and under different physiological conditions. We did not have 24-h monitoring data. Patients with severe conducting disease were excluded, because they had an implanted cardiac device. The definition of first-degree heart block as > 200 ms is arbitrary and for this reason, PR interval duration was used as continuous variable in prognostic models. Due to the observational nature of this study, it is possible that unknown confounding factors may have affected our findings. We necessarily excluded patients with atrial fibrillation. At baseline, atrial fibrillation was common particularly in patients with normal ejection fraction, in whom the prevalence was 36% compared to 5% in patients with HeFREF. This finding is consistent with the previous reports [13] and suggests that atrial fibrillation might be a cause of HeFNEF. Patients with atrial fibrillation might have more severe atrioventricular conduction disease than patients in sinus rhythm, but it will be concealed. If so, we will have underestimated the true prevalence and consequences of conducting system disease in patients with heart failure, especially those with normal ejection fraction.

## Conclusions

A prolonged PR interval is common in patients with chronic heart failure regardless of left ventricular ejection fraction. Although patients with heart failure and a longer PRc have worse survival, PRc is not independently associated with prognosis. Whether PR prolongation is a therapeutic target for pacing therapies in patients with heart failure is currently being tested in randomised trials.

## Electronic supplementary material

Below is the link to the electronic supplementary material.

Supporting Fig. 1 A. Distribution of PRc in 1420 patients with HeFREF. B. Distribution of the change in PRc (ΔPRc) after 1 year in 751 of the 1420 patients with HeFREF. A change in PRc of 50ms means that after adjusting for differences in heart rate, the PR interval increased by 50ms over 1 year C. The number of patients in whom the PRc interval increased, decreased, or stayed at the same quartile (median and interquartile range shown. Numbers outside the boxes represent the number of patients. *498 out of 1420 excluded due to pacemaker implantation, AF, or missing PR value) (JPG 85 KB)

Supporting Fig. 2 Probability of death at 2 years by PR and change in PRc (ΔPRc) at 1 year (N=751). A patient with a baseline PRc of 180ms unchanged at 12 months (Patient A) has a lower probability of death 2 years after first seen than Patient B, whose PRc interval increased from 180ms to 240ms in 12 months (JPG 72 KB)

Supporting Fig. 3 Survival in patients with HeFREF (A), HeFNEF (B) and without heart failure (C) according to baseline PRc and QRS quartile (JPG 78 KB)

Supplementary material 4 (DOC 50 KB)

Supplementary material 5 (DOC 101 KB)

Supplementary material 6 (DOC 106 KB)

Supplementary material 7 (DOC 60 KB)

Supplementary material 8 (DOC 85 KB)

## Abbreviations

CRT: Cardiac resynchronization therapy
eGFR: Estimated glomerular filtraion rate
HeFNEF: Heart failure with normal ejection fraction
HeFREF: Heart failure with reduced ejection fraction
LBBB: Left bundle branch block
LVEF: Left ventricular ejection fraction
NT-ProBNP: Amino-terminal pro-B-type natriuretic peptide
NYHA: New York Heart Association
TSH: Thyroid stimulating hormone
