# Supplementary figures and images for: Prevalence, predictors, and prognostic implications of PR interval prolongation in patients with heart failure
Source: Clin Res Cardiol. 2017 Sep 15;107(2):108–19. doi: 10.1007/s00392-017-1162-6 (PMC5790844; doi:10.1007/s00392-017-1162-6)

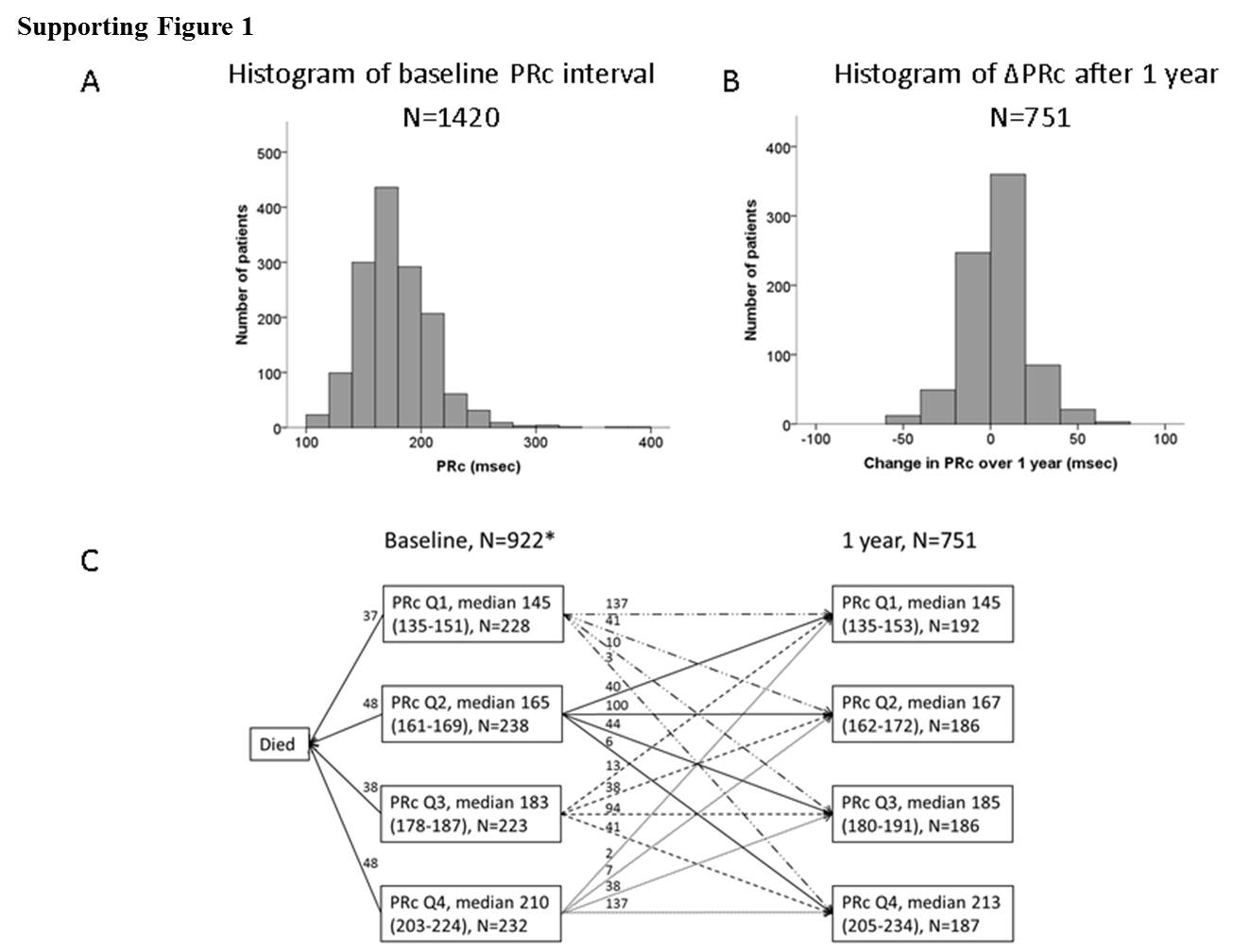

Supplement: Supplementary file 1 — Supporting Fig. 1 A. Distribution of PRc in 1420 patients with HeFREF. B. Distribution of the change in PRc (ΔPRc) after 1 year in 751 of the 1420 patients with HeFREF. A change in PRc of 50ms means that after adjusting for differences in heart rate, the PR interval increased by 50ms over 1 year C. The number of patients in whom the PRc interval increased, decreased, or stayed at the same quartile (median and interquartile range shown. Numbers outside the boxes represent the number of patients. *498 out of 1420 excluded due to pacemaker implantation, AF, or missing PR value) (JPG 85 KB) [file 392_2017_1162_MOESM1_ESM.jpg]

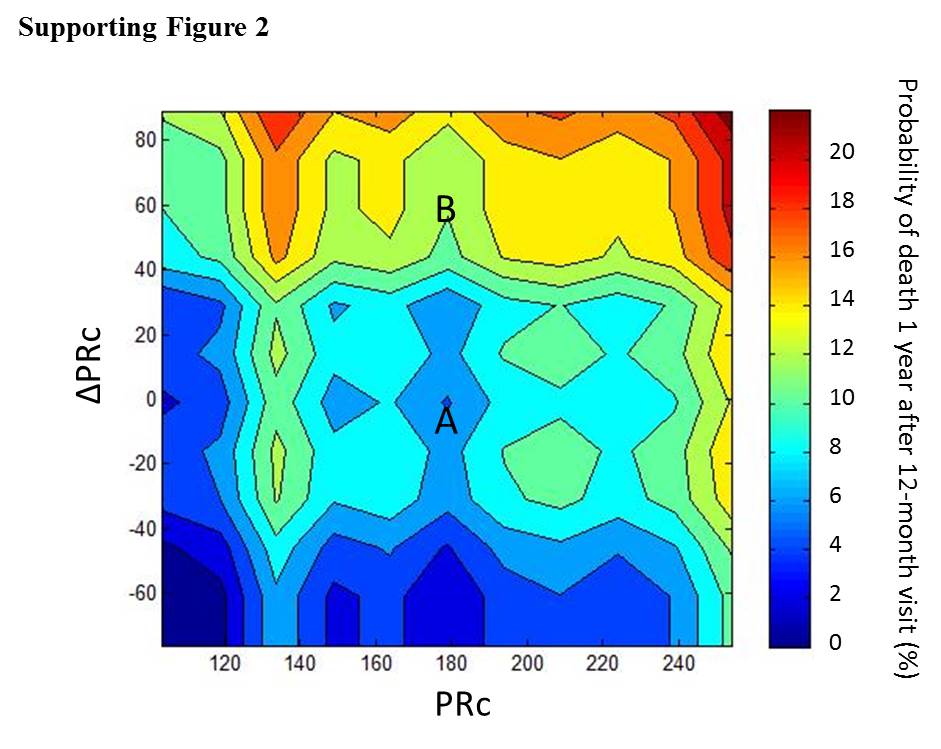

Supplement: Supplementary file 2 — Supporting Fig. 2 Probability of death at 2 years by PR and change in PRc (ΔPRc) at 1 year (N=751). A patient with a baseline PRc of 180ms unchanged at 12 months (Patient A) has a lower probability of death 2 years after first seen than Patient B, whose PRc interval increased from 180ms to 240ms in 12 months (JPG 72 KB) [file 392_2017_1162_MOESM2_ESM.jpg]

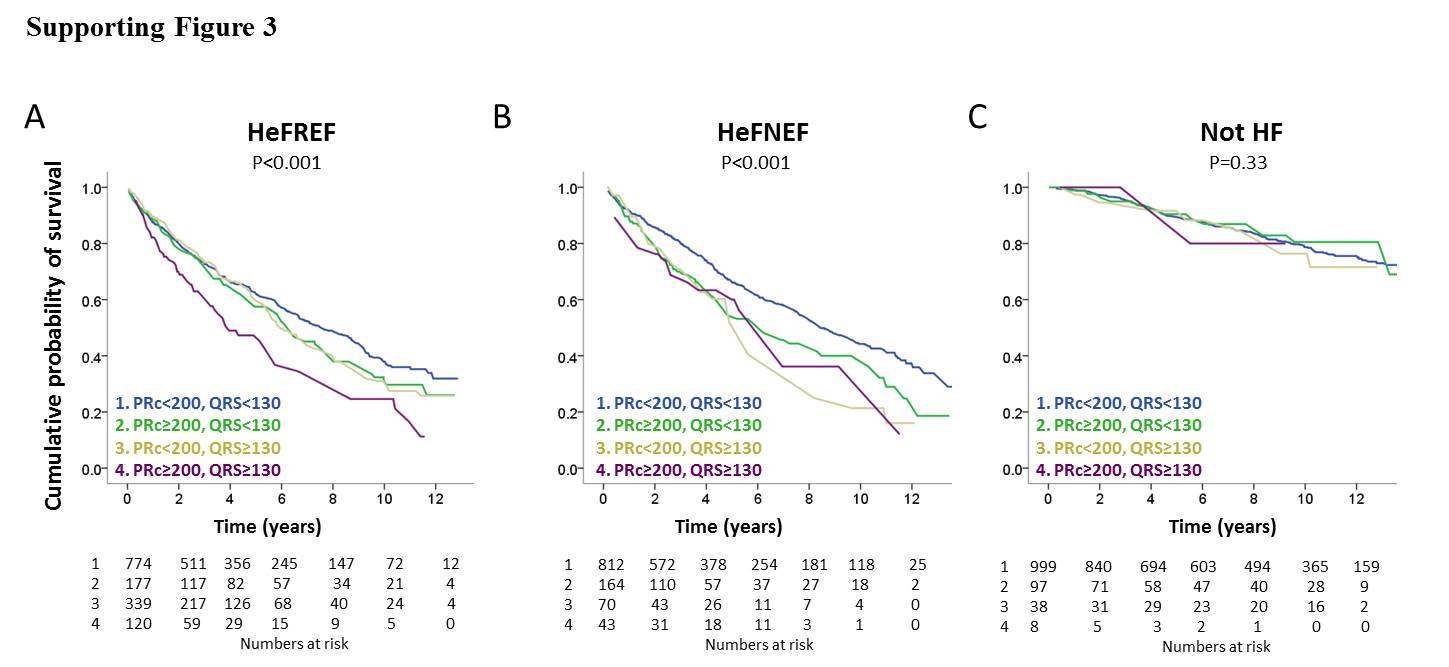

Supplement: Supplementary file 3 — Supporting Fig. 3 Survival in patients with HeFREF (A), HeFNEF (B) and without heart failure (C) according to baseline PRc and QRS quartile (JPG 78 KB) [file 392_2017_1162_MOESM3_ESM.jpg]
