# Supplementary material for: Prevalence, predictors, and prognostic implications of PR interval prolongation in patients with heart failure
Source: Clin Res Cardiol. 2017 Sep 15;107(2):108–19. doi: 10.1007/s00392-017-1162-6 (PMC5790844; doi:10.1007/s00392-017-1162-6)
Supplement: Supplementary file 4 — Supplementary material 4 (DOC 50 KB) [file 392_2017_1162_MOESM4_ESM.doc]

**Supporting Table 3. Bivariate correlations between heart rate, PR, PRc, QRS and QT intervals.**

|  | **HeFREF** | | **HeFNEF** | | **Not HF** | |
| --- | --- | --- | --- | --- | --- | --- |
|  | **Heart rate** | **QRS** | **Heart rate** | **QRS** | **Heart rate** | **QRS** |
| **PR** | R^2^=0.04  P<0.001 | R^2^=0.04  P<0.001 | R^2^=0.03  P<0.001 | R^2^=0.07  P<0.001 | R^2^=0.04  P<0.001 | R^2^=0.04  P<0.001 |
| **PRc** | R^2^<0.001  P=0.79 | R^2^=0.04  P<0.001 | R^2^<0.001  P=0.77 | R^2^=0.07  P<0.001 | R^2^<0.001  P=0.96 | R^2^=0.04  P<0.001 |
| **QRS** | R^2^=0.01  P<0.001 | **-** | R^2^=0.004  P=0.04 | **-** | R^2^=0.02  P=0.001 | **-** |
| **QT** | R^2^=0.29  P<0.001 | R^2^=0.24  P<0.001 | R^2^=0.33  P<0.001 | R^2^=0.11  P<0.001 | R^2^=0.36  P<0.001 | R^2^=0.08  P<0.001 |

Relations between PR, PRc, QRS and QT intervals in patients with HeFREF, HeFNEF and those without heart failure.
