# Supplementary material for: Prevalence, predictors, and prognostic implications of PR interval prolongation in patients with heart failure
Source: Clin Res Cardiol. 2017 Sep 15;107(2):108–19. doi: 10.1007/s00392-017-1162-6 (PMC5790844; doi:10.1007/s00392-017-1162-6)
Supplement: Supplementary file 5 — Supplementary material 5 (DOC 101 KB) [file 392_2017_1162_MOESM5_ESM.doc]

**Supporting Table 2. Baseline demographic and clinical characteristics of patients without heart failure, classified by PRc quartiles.**

|  | **PRc Q1 N=287** | **PRc Q2 N=288** | **PRc Q3 N=288** | **PRc Q4 N=287** | **P-value** |
| --- | --- | --- | --- | --- | --- |
| Age - years | 64 (57-72) | 67 (59-75) | 69 (60-74) | 71 (65-77) | **<0.001** |
| Men - no. (%) | 120 (42) | 129 (45) | 160 (56) | 175 (61) | **<0.001** |
| **NYHA class - no. (%)** |  |  |  |  |  |
| I | 128 (52) | 116 (49) | 117 (48) | 127 (49) | 0.39 |
| II | 86 (35) | 94 (39) | 92 (38) | 97 (37) |
| III | 29 (12) | 24 (10) | 34 (14) | 37 (14) |
| IV | 2 (1) | 4 (2) | 1 (0.4) | 1 (0.4) |
| Diabetes - no. (%) | 50 (17) | 58 (20) | 66 (23) | 86 (30) | **<0.001** |
| Ischaemic heart disease - no. (%) | 64 (22) | 66 (24) | 61 (21) | 55 (19) | 0.36 |
| Cerebrovascular disease - no. (%) | 9 (3) | 13 (5) | 11 (4) | 8 (3) | 0.77 |
| Body surface area - m^2^ | 1.89 (1.73-2.05) | 1.93 (1.74-2.08) | 1.97 (1.81-2.13) | 2.02 (1.84-2.18) | **<0.001** |
| Systolic BP - mmHg | 143 (130-160) | 147 (131-163) | 146 (131-160) | 151 (136-163) | 0.01 |
| Diastolic BP - mmHg | 83 (75-91) | 83 (76-91) | 84 (75-93) | 83 (74-91) | 0.91 |
| Heart rate - bpm | 69 (61-78) | 68 (60-80) | 70 (61-79) | 69 (60-80) | 0.52 |
| QRS - ms | 86 (80-94) | 90 (82-96) | 90 (84-98) | 94 (86-104) | **<0.001** |
| QRS ≥ 150 ms | 3 (1) | 5 (2) | 1 (0.3) | 9 (3) | 0.14 |
| PR - ms | 138 (128-144) | 156 (150-160) | 170 (166-176) | 192 (184-207) | **-** |
| PRc - ms | 138 (129-143) | 155 (151-159) | 170 (167-175) | 194 (185-207) | **-** |
| QT - ms | 394 (366-413) | 396 (370-414) | 396 (373-413) | 400 (378-424) | **0.01** |
| QTc - ms | 414 (400-436) | 417 (400-438) | 419 (402-442) | 425 (405-448) | **0.002** |
| Thyroid stimulating hormone - mIU/L | 1.5 (1.0-2.2) | 1.6 (0.9-2.2) | 1.6 (0.9-2.5) | 1.6 (1.0-2.4) | 0.60 |
| eGFR - 1.73ml/min/m^2^ | 79 (67-89) | 72 (61-86) | 75 (63-88) | 72 (61-84) | **<0.001** |
| NT-ProBNP - ng/l | 83 (45-130) | 93 (51-143) | 83 (42-128) | 89 (47-147) | 0.86 |
| Ejection fraction by Simpson’s | 60 (55-65) | 58 (54-63) | 58 (54-63) | 59 (55-64) | 0.59 |
| **Left ventricular dysfunction - no. (%)** |  |  |  |  | - |
| Normal-Trivial | 287 (100) | 288 (100) | 288 (100) | 287 (100) |
| Mild |  |  |  |  |
| Mild-Moderate | - | - | - | - |
| >Moderate | - | - | - | - |
| **Mitral regurgitation** >mild | 4 (1) | 12 (4) | 9 (3) | 2 (1) | 0.41 |
| β-blocker - no. (%) | 58 (21) | 63 (23) | 75 (28) | 89 (32) | **0.002** |
| ACE-I - no. (%) | 72 (26) | 85 (31) | 74 (27) | 94 (33) | 0.16 |
| ARB - no. (%) | 24 (9) | 29 (11) | 31 (11) | 44 (16) | **0.01** |
| MRA - no. (%) | 1 (0.4) | 7 (3) | 6 (2) | 8 (3) | 0.06 |
| Amiodarone - no. (%) | 1 (0.4) | 1 (0.4) | 3 (1) | 1 (0.4) | 0.73 |
| Digoxin - no. (%) | 0 | 2 (1) | 4 (1) | 3 (1) | 0.11 |
| Loop Diuretic - no. (%) | 53 (19) | 67 (23) | 62 (22) | 67 (23) | 0.26 |
| Ivabradine - no. (%) | 0 | 0 | 0 | 0 | - |
| 1 year mortality | 2 (1) | 4 (1) | 1 (0.3) | 1 (0.3) | 0.34 |

Continuous variables are presented as median (interquartile range), whereas categorical variables are expressed as numbers (percentage). P-values are for differences between PRc quartiles (columns 2, 3, 4 and 5). The one-way ANOVA linear trend test was used for comparisons of continuous data across groups and the Cochran’s chi-square trend test for categorical data. ACE-I, angiotensin converting enzyme inhibitor; ARB, angiotensin receptor blocker; BP, blood pressure; eGFR, estimated glomerular filtration rate; MRA, mineralocorticoid receptor antagonist; NYHA, New York Heart Association.
