# Supplementary material for: Prevalence, predictors, and prognostic implications of PR interval prolongation in patients with heart failure
Source: Clin Res Cardiol. 2017 Sep 15;107(2):108–19. doi: 10.1007/s00392-017-1162-6 (PMC5790844; doi:10.1007/s00392-017-1162-6)
Supplement: Supplementary file 6 — Supplementary material 6 (DOC 106 KB) [file 392_2017_1162_MOESM6_ESM.doc]

**Supporting Table 1. Baseline demographic and clinical characteristics of patients with HeFNEF, classified by PRc quartiles.**

|  | **PRc Q1 N=274** | **PRc Q2 N=274** | **PRc Q3 N=273** | **PRc Q4 N=273** | **P-value** |
| --- | --- | --- | --- | --- | --- |
| Age - years | 75 (68-80) | 76 (69-81) | 76 (70-82) | 78 (72-84) | **<0.001** |
| Men - no. (%) | 98 (36) | 118 (43) | 129 (47) | 171 (63) | **<0.001** |
| **NYHA class - no. (%)** |  |  |  |  |  |
| I | 92 (35) | 82 (30) | 83 (31) | 68 (25) | **0.04** |
| II | 114 (43) | 123 (45) | 133 (49) | 123 (46) |
| III | 54 (20) | 62 (23) | 51 (19) | 77 (28) |
| IV | 5 (2) | 4 (2) | 3 (1) | 3 (1) |
| Diabetes - no. (%) | 68 (25) | 62 (22) | 86 (31) | 75 (27) | 0.14 |
| Ischaemic heart disease - no. (%) | 114 (42) | 124 (45) | 123 (45) | 118 (43) | 0.66 |
| Cerebrovascular disease - no. (%) | 16 (6) | 22 (8) | 18 (7) | 12 (4) | 0.39 |
| Body surface area - m^2^ | 1.81 (1.65-2.01) | 1.84 (1.70-1.99) | 1.85 (1.71-2.03) | 1.94 (1.77-2.11) | **<0.001** |
| Systolic BP - mmHg | 150 (133-167) | 149 (130-168) | 150 (133-167) | 149 (130-169) | 0.85 |
| Diastolic BP - mmHg | 77 (70-89) | 78 (69-89) | 78 (70-88) | 78 (70-87) | 0.56 |
| Heart rate - bpm | 67 (59-77) | 68 (59-78) | 67 (59-79) | 68 (60-80) | 0.45 |
| QRS - ms | 88 (80-98) | 90 (82-102) | 94 (84-104) | 100 (89-121) | **<0.001** |
| QRS ≥ 150 ms | 6 (2) | 14 (5) | 8 (3) | 25 (9) | **0.001** |
| PR - ms | 140 (132-146) | 160 (154-166) | 180 (172-186) | 214 (200-238) | **-** |
| PRc - ms | 140 (131-147) | 159 (155-164) | 178 (174-185) | 212 (201-236) | **-** |
| QT - ms | 402 (380-426) | 407 (385-432) | 408 (384-429) | 408 (384-438) | **0.01** |
| QTc - ms | 424 (407-446) | 428 (408-452) | 428 (409-452) | 436 (415-460) | **0.001** |
| Thyroid stimulating hormone - mIU/L | 1.6 (0.9-2.6) | 1.7 (1.1-2.4) | 1.7 (1.2-2.4) | 1.7 (1.1-2.7) | 0.12 |
| eGFR - 1.73ml/min/m^2^ | 62 (49-76) | 59 (46-73) | 64 (49-79) | 57 (42-72) | **0.04** |
| NT-ProBNP - ng/l | 464 (294-1024) | 495 (325-1229) | 507 (325-1278) | 643 (368-1595) | 0.13 |
| Ejection fraction by Simpson’s | 55 (49-61) | 55 (48-62) | 54 (49-60) | 52 (46-59) | **0.02** |
| **Left ventricular dysfunction - no. (%)** |  |  |  |  | 0.06 |
| Normal/Trivial | 211 (77) | 213 (77) | 203 (74) | 193 (71) |
| Mild | 63 (23) | 61 (23) | 70 (26) | 80 (29) |
| Mild-Moderate | 0 | 0 | 0 | 0 |
| >Moderate | 0 | 0 | 0 | 0 |
| **Mitral regurgitation** >mild | 25 (11) | 28 (12) | 38 (16) | 34 (14) | 0.19 |
| β-blocker - no. (%) | 120 (46) | 144 (53) | 134 (50) | 152 (56) | 0.03 |
| ACE-I - no. (%) | 112 (42) | 148 (54) | 134 (50) | 138 (51) | 0.12 |
| ARB - no. (%) | 34 (13) | 46 (17) | 37 (14) | 44 (16) | 0.46 |
| MRA - no. (%) | 23 (8) | 22 (8) | 23 (8) | 27 (10) | 0.57 |
| Amiodarone - no. (%) | 4 (2) | 3 (1) | 3 (1) | 9 (3) | 0.13 |
| Digoxin - no. (%) | 6 (2) | 9 (3) | 9 (3) | 16 (6) | **0.03** |
| Loop Diuretic - no. (%) | 119 (45) | 143 (52) | 139 (52) | 156 (58) | **0.01** |
| Ivabradine - no. (%) | 1 (0.4) | 0 | 1 (0.4) | 0 | 0.52 |
| 1 year mortality | 16 (6) | 21 (8) | 16 (6) | 23 (8) | 0.39 |

Continuous variables are presented as median (interquartile range), whereas categorical variables are expressed as numbers (percentage). P-values are for differences between PRc quartiles (columns 2, 3, 4 and 5). The one-way ANOVA linear trend test was used for comparisons of continuous data across groups and the Cochran’s chi-square trend test for categorical data. ACE-I, angiotensin converting enzyme inhibitor; ARB, angiotensin receptor blocker; BP, blood pressure; eGFR, estimated glomerular filtration rate; MRA, mineralocorticoid receptor antagonist; NYHA, New York Heart Association.
