# Supplementary material for: Prevalence, predictors, and prognostic implications of PR interval prolongation in patients with heart failure
Source: Clin Res Cardiol. 2017 Sep 15;107(2):108–19. doi: 10.1007/s00392-017-1162-6 (PMC5790844; doi:10.1007/s00392-017-1162-6)
Supplement: Supplementary file 7 — Supplementary material 7 (DOC 60 KB) [file 392_2017_1162_MOESM7_ESM.doc]

**Supporting Table 5. Hazard ratios for pacemaker implantation from baseline characteristics in patients with HeFREF, HeFNEF and those without heart failure.**

| **Cox Regression** |  | **HeFREF N=1388** | |  | **HeFNEF N=1069** | | | **Not HF N=985** | | |
| --- | --- | --- | --- | --- | --- | --- | --- | --- | --- | --- |
| **Variable** | **Hazard ratio represents** | **Hazard Ratio**  **(95% CI)** | **P-value** | **Wald** | **Hazard Ratio**  **(95% CI)** | **P-value** | **Wald** | **Hazard Ratio**  **(95% CI)** | **P-value** | **Wald** |
| QRS | Per 10 ms increase | 1.20 (1.07, 1.24) | **0.001** | 10.13 | 1.03 (0.92, 1.16) | 0.60 | 0.28 | 1.24 (1.01, 1.52) | **0.04** | 4.33 |
| PRc | Per 10 ms increase | 1.13 (1.05, 1.21) | **0.002** | 10.07 | 1.14 (1.07, 1.22) | **<0.001** | 16.64 | 1.19 (1.09, 1.28) | **<0.001** | 19.05 |
| Age | Per decade increase | 1.80 (1.16, 2.77) | **0.01** | 7.01 | 0.94 (0.63, 1.40) | 0.76 | 0.09 | 1.56 (0.89, 2.73) | 0.12 | 2.41 |
| NYHA | III/IV vs. I/II | 1.75 (0.89, 3.42) | 0.10 | 2.64 | 1.99 (0.97, 4.09) | 0.06 | 3.71 | 1.71 (0.49, 5.97) | 0.40 | 0.70 |
| eGFR | Per 5 ml/min/1.73m^2^ increase | 0.99 (0.89, 1.09) | 0.82 | 0.05 | 0.92 (0.84, 1.00) | 0.05 | 3.83 | 0.99 (0.84, 1.18) | 0.96 | 0.003 |

Variables ranked by Wald score. Implantable defibrillators and CRT devices have been excluded.
