# Supplementary material for: Prevalence, predictors, and prognostic implications of PR interval prolongation in patients with heart failure
Source: Clin Res Cardiol. 2017 Sep 15;107(2):108–19. doi: 10.1007/s00392-017-1162-6 (PMC5790844; doi:10.1007/s00392-017-1162-6)
Supplement: Supplementary file 8 — Supplementary material 8 (DOC 85 KB) [file 392_2017_1162_MOESM8_ESM.doc]

**Supporting Table 4: Baseline and 1 year demographic and clinical characteristics of patients with HeFREF.**

|  |  | **Baseline**  **N=751** | **1 year**  **N=751** | **P-value** |
| --- | --- | --- | --- | --- |
| Age - years |  | 70 (62-76) | 71 (63-77) | - |
| Men - no. (%) |  | 538 (72) | | - |
| **NYHA class -no. (%)** | 0/4 |  |  |  |
| I |  | 137 (18) | 175 (23) | **<0.001** |
| II |  | 386 (51) | 419 (56) |  |
| III |  | 211 (28) | 144 (19) |  |
| IV |  | 17 (2) | 9 (1) |  |
| Body surface area - m^2^ |  | 1.92 (1.76-2.09) | 1.92 (1.75-2.09) | 0.32 |
| Systolic BP - mmHg | 5/19 | 130 (116-147) | 127 (113-142) | **0.002** |
| Diastolic BP - mmHg | 5/19 | 76 (66-85) | 72 (64-80) | **<0.001** |
| HR - bpm | 0/1 | 71 (60-83) | 65 (58-74) | **<0.001** |
| QRS - ms | 2/0 | 110 (96-140) | 112 (98-142) | **0.003** |
| QRS ≥ 150 ms |  | 136 (18) | 140 (19) | 0.59 |
| PR - ms |  | 172 (154-194) | 178 (160-200) | **<0.001** |
| PRc - ms |  | 173 (156-193) | 176 (158-197) | **<0.001** |
| DPR - ms |  |  | 3.30 (-6.32-12.72) |  |
| QT - ms | 10/1 | 418 (388-450) | 424 (396-454) | **<0.001** |
| QTc - ms | 10/2 | 447 (417-483) | 437 (417-467) | **<0.001** |
| eGFR - 1.73ml/min/m^2^ | 0/52 | 63 (48-76) | 58 (42-72) | **<0.001** |
| NT-ProBNP - ng/l | 192/181 | 1136 (504-2926) | 761 (354-1793) | **<0.001** |
| **Left ventricular systolic dysfunction - no. (%)** | 0/42 |  |  |  |
| Normal-Trivial |  | 0 | 49 (7) | **<0.001** |
| Mild |  | 1 (<1) | 122 (17) |  |
| Mild-moderate |  | 450 (60) | 342 (48) |  |
| >Moderate |  | 300 (40) | 196 (28) |  |
| Ejection fraction by Simpson’s | 265/371 | 32 (26-37) | 35 (30-43) | **<0.001** |
| β-blocker - no. (%) | 2/0 | 458 (61) | 652 (87) | **0.001** |
| ACE-I - no. (%) | 2/16 | 565 (75) | 582 (78) | **0.02** |
| ARB - no. (%) | 3/18 | 63 (8) | 112 (15) | **<0.001** |
| MRA - no. (%) | 0/16 | 206 (28) | 278 (37) | **<0.001** |
| Amiodarone - no. (%) | 0/16 | 35 (5) | 35 (5) | 1.00 |
| Digoxin - no. (%) | 0/16 | 61 (8) | 79 (11) | **0.01** |
| Loop diuretic - no. (%) | 2/2 | 542 (72) | 557 (74) | 0.17 |
| Ivabradine | 2/16 | 0 | 9 (1) | **0.004** |

Continuous variables are presented as median (interquartile range), whereas categorical variables are expressed as numbers (percentage). P-values are for differences between baseline and 1 year. The paired t-test was used for continuous data (except for NT-ProBNP where the Wilcoxon signed rank test was used). For categorical data with two or more categories the McNemar’s test and the marginal homogeneity non-parametric test were used, respectively. ACE-I, angiotensin converting enzyme inhibitor; ARB, angiotensin receptor blocker; BP, blood pressure; eGFR, estimated glomerular filtration rate; MRA, mineralocorticoid receptor antagonist; NYHA, New York Heart Association.
